# Errata

**Published:** 2005-04

Lai et al. would like to clarify their financial support for their article “Understanding the Spatial Clustering of Severe Acute Respiratory Syndrome (SARS) in Hong Kong” [ Environ Health Perspect 112:1550–1556 (2004)]. The acknowledgment should have stated that “The research was supported by the Research Fund for the Control of Infectious Diseases of the Health, Welfare and Food Bureau of the Hong Kong SAR Government.”

The January Beyond the Bench article “Building Blocks of Learning” [ Environ Health Perspect 113:A33 (2005)] incorrectly listed Kathleen Vandiver's affiliation as Cambridge Public Schools. Vandiver is actually with Lexington Public Schools.

The March Science Selections article “Fewer Frogs in Illinois: Organochlorines May Be to Blame” [ Environ Health Perspect 113:A182 (2005)] listed incorrect percentages of intersex frogs collected during the periods studied. In fact, intersex frogs accounted for 1.2% of samples from 1852 to 1929, 7.5% of samples from 1930 to 1945, 11.1% of samples from 1946 to 1959, 6.3% of samples from 1960 to 1979, and 2.7% of samples from 1980 to 2001.

*EHP* regrets the errors.

